# The Potential Role of MiRs-139-5p and -454-3p in Endoglin-Knockdown-Induced Angiogenic Dysfunction in HUVECs

**DOI:** 10.3390/ijms24054916

**Published:** 2023-03-03

**Authors:** Anthony Cannavicci, Qiuwang Zhang, Michael J. B. Kutryk

**Affiliations:** 1 Institute of Medical Science, University of Toronto, Toronto, ON M5S 1A8, Canada; 2 Division of Cardiology, Keenan Research Center for Biomedical Science, St. Michael’s Hospital, Unity Health Toronto, Toronto, ON M5B 1T8, Canada

**Keywords:** hereditary hemorrhagic telangiectasia, endoglin, microRNA, endothelial cell, angiogenesis

## Abstract

Hereditary hemorrhagic telangiectasia (HHT) is a rare genetic disease characterized by aberrant angiogenesis and vascular malformations. Mutations in the transforming growth factor beta co-receptor, endoglin (*ENG*), account for approximately half of known HHT cases and cause abnormal angiogenic activity in endothelial cells (ECs). To date, how ENG deficiency contributes to EC dysfunction remains to be fully understood. MicroRNAs (miRNAs) regulate virtually every cellular process. We hypothesized that ENG depletion results in miRNA dysregulation that plays an important role in mediating EC dysfunction. Our goal was to test the hypothesis by identifying dysregulated miRNAs in *ENG*-knockdown human umbilical vein endothelial cells (HUVECs) and characterizing their potential role in EC function. We identified 32 potentially downregulated miRNAs in *ENG*-knockdown HUVECs with a TaqMan miRNA microarray. MiRs-139-5p and -454-3p were found to be significantly downregulated after RT-qPCR validation. While the inhibition of miR-139-5p or miR-454-3p had no effect on HUVEC viability, proliferation or apoptosis, angiogenic capacity was significantly compromised as determined by a tube formation assay. Most notably, the overexpression of miRs-139-5p and -454-3p rescued impaired tube formation in HUVECs with *ENG* knockdown. To our knowledge, we are the first to demonstrate miRNA alterations after the knockdown of *ENG* in HUVECs. Our results indicate a potential role of miRs-139-5p and -454-3p in *ENG*-deficiency-induced angiogenic dysfunction in ECs. Further study to examine the involvement of miRs-139-5p and -454-3p in HHT pathogenesis is warranted.

## 1. Introduction

Hereditary hemorrhagic telangiectasia (HHT) is a rare genetic disease inherited in an autosomal dominant fashion that can lead to life-threatening vascular dysplasia. Approximately 1 in 5000 to 8000 people are affected globally [1]. HHT patients can develop vascular malformations that form a direct connection between arteries and veins absent of capillaries, called telangiectasias and arteriovenous malformations (AVMs) [2,3]. Telangiectasias are superficial dilated blood vessels that form on the skin and mucocutaneous tissue [4]. Approximately 95% of patients with HHT develop epistaxis due to nasal telangiectasias [5]. AVMs are greater in size and can develop in various locations, including the lungs, brain, liver and spine [6]. Severe complications can arise from untreated AVMs, including hypoxia, brain abscess, high-output cardiac heart failure, hypertension and ischemic and hemorrhagic stroke [6]. There is no cure for HHT, and effective pharmacological therapies are limited.

It was found that mutations in three genes cause HHT, including endoglin (*ENG*, chromosomal locus 9q34) [7], activin-receptor like kinase 1 (*ACVRL1*, also known as *ALK1*, chromosomal locus 12q1) [8] and mothers against decapentaplegic homolog 4 (*SMAD4*, chromosomal locus 18q21) [9]. Additionally, mutations in growth/differentiation factor 2 (*GDF2*, chromosomal locus 10q11) [10] and Ras p21 protein activator 1 (*RASA1*, chromosomal locus 5q14) [10] create HHT-like syndromes. *ENG* and *ACVRL1* mutations are responsible for approximately 70–90% of confirmed HHT cases and lead to HHT Type 1 and 2, respectively [11,12,13]. *SMAD4* mutations are responsible for approximately 1–2% of HHT cases and can result in a combined juvenile polyposis-HHT syndrome (JP-HHT) [14]. There are over 850 known disease-causing mutations in *ENG, ACVRL1* and *SMAD4* (https://arup.utah.edu/database/HHT/, https://arup.utah.edu/database/SMAD4/SMAD4_welcome.php, access date: 3 November 2021) that most commonly include missense mutations, although single base pair changes, large deletions, duplications, frameshifts and substitutions have also been documented [15].

*ENG*, *ACVRL1*, *SMAD4* and *GDF2* are all involved in the transforming growth factor beta/bone morphogenetic protein (TGFβ/BMP) signaling pathway, while *RASA1* predominately regulates PI3K/Akt signaling [16]. These pathways are integral in the regulation of various cellular processes, including growth, differentiation, apoptosis and, importantly, endothelial cell (EC) function and angiogenesis. The pathogenic role of these mutations has been confirmed in mouse models, where the loss of *ENG*, *ACVRL1* or *SMAD4* results in various vascular defects [17,18]. *ACVRL1* encodes for a TGFβ receptor I that is mostly expressed on endothelial, lung and placental cells. *SMAD4* is a downstream effector of the TGFβ/BMP signaling pathway that upon activation translocates to the nucleus to regulate gene expression. *ENG*, predominantly expressed in the endothelium, activated monocytes and macrophages, is a co-receptor that ensures a high-affinity bond between ligands and TGFβ receptors I/II.

MicroRNAs (miRNAs) are short non-coding RNA molecules, approximately 21–25 nucleotides long, that regulate gene expression in a post-transcriptional manner [19]. Since their discovery in 1993 by the Ambros and Ruvkin groups, over 2000 miRNAs have been identified [20,21]. MiRNAs have been shown to be involved in a vast array of cellular processes regulating approximately 30% of known genes [22,23,24]. Processed in the nucleus and cytoplasm by endoribonucleases, such as RNAase III, they exert their effects through the interaction with the 3′ untranslated region (UTR) of messenger RNA (mRNA) [25]. Typically, human miRNAs bind imperfectly and silence mRNAs through the blockage of translational machinery [22]. These low-fidelity molecules have been shown to target tens to hundreds of genes, while groups of miRNAs expressed from the same transcript, known as clusters or families, share similar target homology [22,26]. MiRNAs are involved in almost every cellular process and have been implicated in the pathogenesis of human diseases [27]. They have been shown to be reliable biomarkers, especially in oncology, and are being investigated as novel therapeutic targets [28,29].

Endoglin (*ENG*) is primarily expressed in endothelial cells (ECs), and loss of *ENG* has been shown in numerous studies to result in abnormal angiogenic function in ECs [30]. However, how ENG deficiency contributes to EC dysfunction remains to be fully understood. We hypothesized that ENG depletion results in miRNA dysregulation that contributes to EC dysfunction. To test this hypothesis, we performed a miRNA microarray analysis and RT-qPCR to identify dysregulated miRNAs in *ENG*-knockdown human umbilical vein endothelial cells (HUVECs) and further characterize their potential role in EC function.

## 2. Results

The N shown in all figures is the number of independent experiments.

### 2.1. ENG-Knockdown HUVECs Demonstrated a Potentially Dysregulated MiRNA Profile

**Figure 1:**
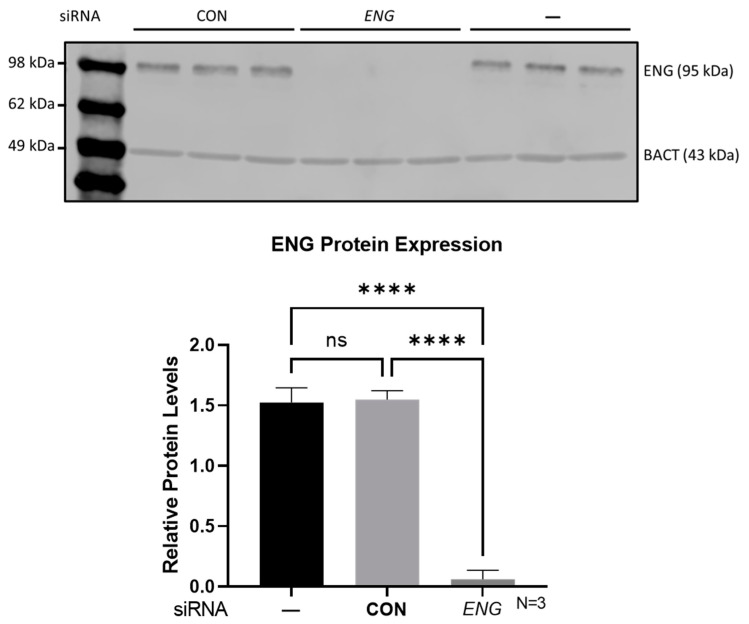
*ENG* siRNA (*ENG*) effectively depleted ENG protein in HUVECs compared with that in negative control siRNAs (CON) and non-transfected controls (—) shown via Western blot analysis (upper panel). Bar graph represents relative ENG protein levels (lower panel). **** *p* < 0.0001, ns: not significant (*p* = 0.979).

**Figure 2:**
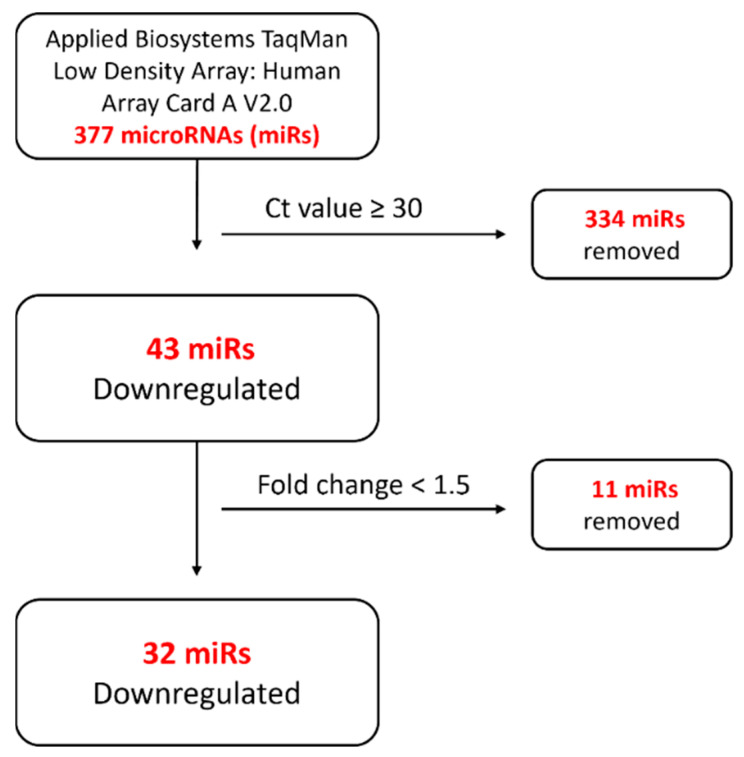
Systematic miRNA exclusion flow chart of miRNA microarray analysis. Ct: cycle threshold.

*ENG*-siRNA transfection significantly depleted ENG protein in HUVECs compared with that in non-transfected and control siRNA-transfected HUVECs as shown by Western blot analysis (Figure 1). A TaqMan miRNA microarray with 377 human miRNA targets was employed to identify potentially dysregulated miRNAs in *ENG*-knockdown HUVECs compared with negative control siRNA HUVECs. MiRNAs that had less than a 1.5-fold change and a cycle threshold (Ct) value ≥ 30 were systematically excluded (Figure 2). Three independent miRNA microarray analyses were performed for *ENG*-knockdown and control HUVECs, that identified a total of 32 miRNAs as potentially downregulated (Table 1) and none as upregulated in *ENG*-knockdown HUVECs.

### 2.2. Significantly Reduced Levels of MiRs-139-5p and -454-3p in ENG-Knockdown HUVECs

**Figure 3:**
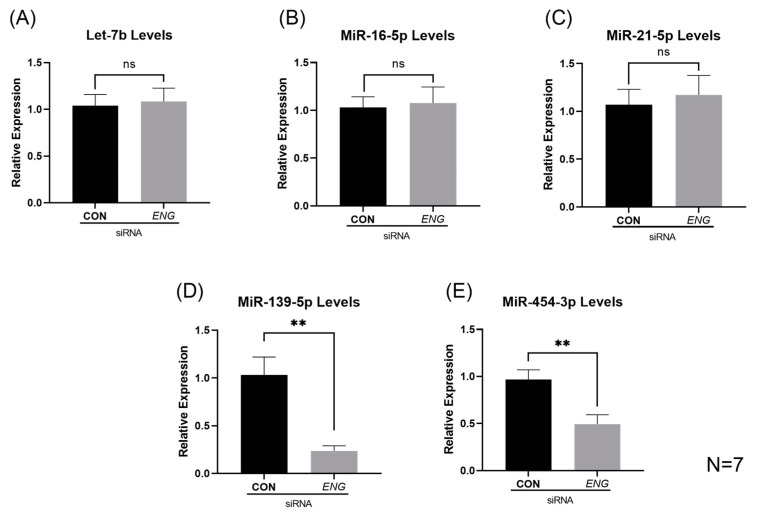
RT-qPCR validation of miRs-let-7b, -16-5p, -21-5p, -139-5p and -454-3p. (**A**–**C**) MiRs-let-7b, -16-5p and -21-5p were not found to be significantly decreased in *ENG*-knockdown HUVECs. (**D**,**E**) MiRs-139-5p and -454-3p were found to be significantly decreased in *ENG*-knockdown HUVECs compared with those in negative control siRNA HUVECs. ns: not significant, ** p < 0.01.

The MicroRNA Microarray Card A v2.0 used in this study detects both non-angiogenic and angiogenic miRNAs. Of the 32 potentially dysregulated miRNAs identified by the array analysis, those with unknown EC angiogenic activity, i.e., miR-99a-5p, miR-99b-5p and miR-574-3p, were not chosen for further study. MiRNAs, whose downregulation has been documented in the literature to promote tube formation in ECs, which is discordant with angiogenic dysfunction seen in ENG-deficient ECs, were not characterized further either, such as miR-191-5p, miR-125b-5p, miR-31-5p, etc. [31,32,33]. MiR-126-3p, one of the best-characterized angiogenic miRNAs in ECs [34], was not investigated further, as a previous study showed that ENG depletion does not affect the target genes of miR-126-3p in HUVECs [35]. Eventually, miRs-let-7b, -16-5p, -21-5p, -139-5p and -454-3p were selected for RT-qPCR validation. As shown in Figure 3, miRs-139-5p and -454-3p were significantly reduced (*p* = 0.0048 and *p* = 0.0062, respectively) in *ENG*-knockdown HUVECs, while the levels of miRs-let-7b, -16-5p and -21-5p were not significantly different as compared with those in controls. MiR-139-5p demonstrated a 4.4-fold decrease, and miR-454-3p demonstrated a 2-fold decrease in *ENG*-knockdown HUVECs, respectively.

### 2.3. Inhibition of MiR-139-5p or MiR-454-3p Had No Effect on HUVEC Viability and Proliferation

**Figure 4:**
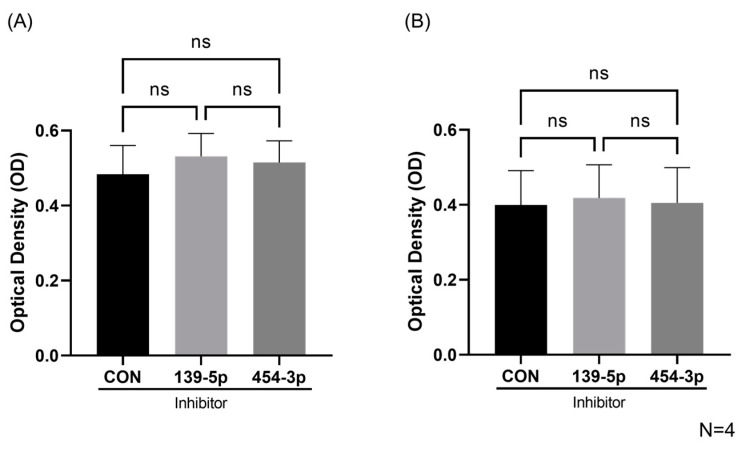
Viability and proliferation of HUVECs with miR-139-5p or miR-454-3p inhibition. (**A**) No significant differences were found in OD for HUVEC viability with miR-139-5p or miR-454-3p inhibitors compared with negative control inhibitors (CON) (N = 4). (**B**) No significant differences were found in OD for HUVEC proliferation with miR-139-5p or miR-454-3p inhibitors compared with negative control inhibitors (CON) (N = 4). ns: not significant.

In the context of HHT, the literature demonstrates that HHT Type 1 (ENG-deficient) ECs have increased rates of proliferation and viability [30]. To understand the role miRs-139-5p and -454-3p may play in HUVEC function, we inhibited these miRNAs individually and assessed HUVEC viability and proliferation with a CCK8 assay. MiRs-139-5p and -454-3p were both successfully downregulated after miRNA inhibition as shown in Supplementary Figure S2. The CCK8 assay demonstrated that the inhibition of miR-139-5p or miR-454-3p had no effect on either HUVEC viability or proliferation (Figure 4A,B, respectively). For viability, the inhibition of miRs-139-5p or -454-3p returned an OD of 0.53 ± 0.12 and 0.51 ± 0.11, respectively, compared to the negative control’s OD of 0.48 ± 0.14. In terms of proliferation, the inhibition of miRs-139-5p or -454-3p returned an OD of 0.41 ± 0.16 and 0.40 ± 0.17, respectively, compared to the negative control’s OD of 0.40 ± 0.16.

### 2.4. Inhibition of MiR-139-5p or MiR-454-3p Had No Effect on HUVEC Apoptosis

**Figure 5:**
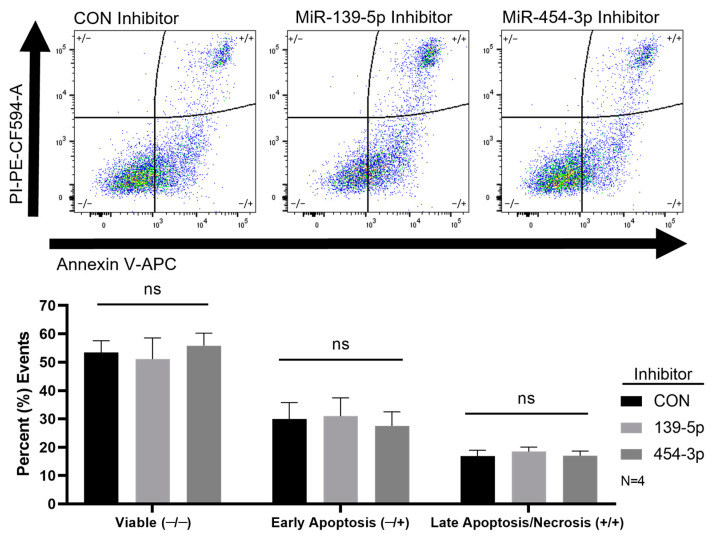
Annexin V and PI staining of HUVECs assessed by flow cytometry. The upper panel shows representative dot plots of Annexin V and PI staining of different groups of cells. No significant differences in the percent of cellular events were found for HUVEC viability, early apoptosis and late apoptosis/necrosis with miR-139-5p or miR-454-3p inhibition compared with negative control inhibitors (lower panel, N = 4). ns: not significant.

Apoptotic events, determined by the flow cytometric detection of AV, were unchanged when miR-139-5p or miR-454-3p were inhibited in HUVECs compared with those in negative controls (Figure 5). The percentages of double-stained (+/+) or AV+/PI+ events or late apoptotic/necrotic cells were 16.8 ± 4.22, 18.45 ± 3.20 and 16.97 ± 3.325 for negative controls, miR-139-5p inhibition and miR-454-3p inhibition, respectively (Figure 5). The percentages of AV-stained (−/+) or PI−/AV+ events or early apoptotic cells were 29.95 ± 11.54, 30.92 ± 13.00 and 27.47 ± 9.99 for negative controls, miR-139-5p inhibition and miR-454-3p inhibition, respectively (Figure 5). Necrotic cells or only PI-stained events were barely detectable.

### 2.5. Inhibition of MiR-139-5p Augmented HUVEC Migration

**Figure 6:**
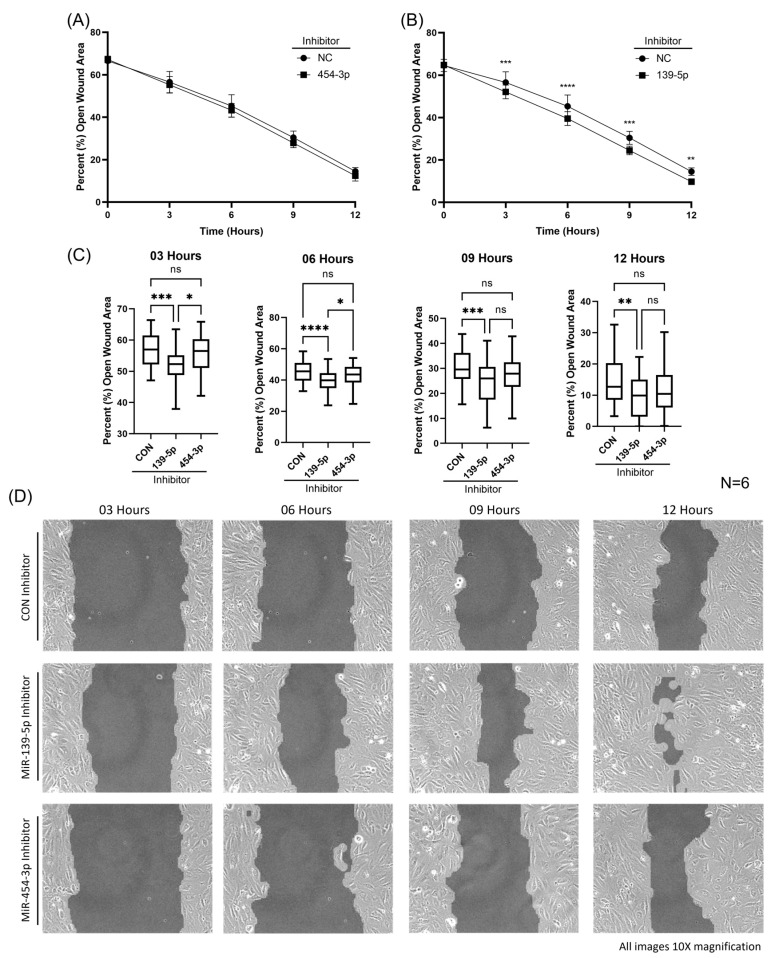
Measurement of HUVEC migration rates with an Ibidi wound healing assay. (**A**) The inhibition of miR-454-3p had no effect on HUVEC migration as shown by the percentage (%) of open wound area compared with that in negative controls (N = 6). (**B**) The inhibition of miR-139-5p resulted in significantly increased rates of migration as determined by the percentage (%) of open wound area compared with that in negative controls (N = 6). (**C**) Box plots display significant differences in HUVEC migration rates with miR-139-5p inhibition at 3, 6, 9 and 12 h measured by percentage (%) of open wound area. (**D**) Images of HUVEC migration with miR-139-5p inhibition, miR-454-3p inhibition and negative control inhibitors at 3, 6, 9 and 12 h (10× magnification). ns: not significant, * *p* < 0.05, ** *p* < 0.01, *** *p* < 0.001, **** *p* < 0.0001.

It has been well established in both in vitro and in vivo models that the loss of ENG results in perturbed EC migration [36,37,38]. To further understand the role miR-139-5p or miR-454-3p plays in EC function, we assessed cell migration with an Ibidi wound healing assay. The inhibition of miR-454-3p in HUVECs had no effect on migration rates compared with that in negative controls shown in Figure 6A. Interestingly, the inhibition of miR-139-5p resulted in significantly increased rates of migration (Figure 6B), as determined by the percentage (%) of open wound area, at 3, 6, 9 and 12 h compared with that in negative controls (Figure 6C). No significant differences in the percentage of open wound area were found at the start of the assay (0 h) between the miRNA inhibitor- and control-transfected HUVECs (Figure 6A,B). Images of cell migration among groups at 3, 6, 9 and 12 h are shown in Figure 6D.

### 2.6. Reduction of MiR-139-5p or MiR-454-3p Impaired Tube Formation of HUVECs In Vitro

**Figure 7:**
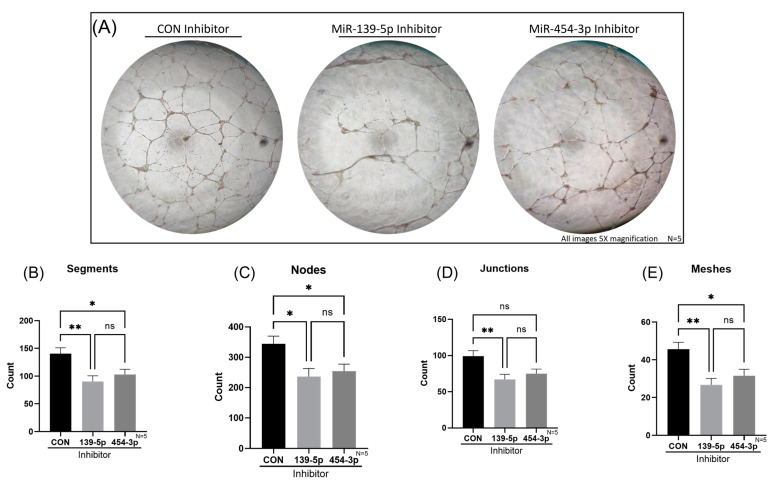
In vitro tube formation assay of HUVECs with miR-139-5p or miR-454-3p inhibition compared with that of negative controls (CON). (**A**) Images taken at 16 h of negative control, miR-139-5p inhibition and miR-454-3p inhibition HUVEC tube formation (5× magnification). (**B**,**C**,**E**) Significantly lower counts of segments, nodes and meshes were detected for HUVECs with miR-139-5p or miR-454-3p inhibition compared with those in negative controls. (**D**) Significantly lower counts of junctions were found for the inhibition of miR-139-5p, while the inhibition of miR-454-3p returned lower counts of junctions, but it was not statistically significant (N = 5). ns: not significant, * *p* < 0.05, ** *p* < 0.01.

The role of *ENG* in angiogenesis has been well established in the general literature [30] as well as in the context of HHT, especially in mouse models [17]. However, to our knowledge, only Fernandez-L et al. have demonstrated deficient in vitro tube formation of HHT Type 1 and 2 blood outgrowth ECs (BOECs) [39]. Interestingly, they determined that the reduced ability of tube formation is correlated with reduced *ENG* expression in both HHT Type 1 and 2 BOECs [39]. Whether miRs-139-5p and -454-3p contribute to EC dysfunction in this regard remains to be clarified. We investigated how the inhibition of miR-139-5p or miR-454-3p affected the angiogenic capacity of HUVECs with an in vitro tube-formation assay. A significant reduction of tube formation was observed for the inhibition of miR-139-5p or miR-454-3p compared with that in negative controls based on four parameters: segments, nodes, junctions and meshes (Figure 7A). A detailed description of these parameters can be found in Supplementary Figure S3. In brief, segments refer to connected tubes, nodes are central connecting points, junctions are points with three or more connecting segments and meshes are complete ring structures. The number of segments was 90 ± 32 (*p* < 0.01), 103 ± 30 (*p* < 0.05) and 141 ± 33 for miR-139-5p inhibition, miR-454-3p inhibition and negative controls, respectively (Figure 7B). The number of nodes was 237 ± 83 (*p* < 0.05), 254 ± 73 (*p* < 0.05) and 344 ± 80 for miR-139-5p inhibition, miR-454-3p inhibition and negative controls, respectively (Figure 7C). The number of junctions was 67 ± 22 (*p* < 0.01), 75 ± 20 and 99 ± 23 for miR-139-5p inhibition, miR-454-3p inhibition and negative controls, respectively (Figure 7D). Lastly, the number of meshes was 27 ± 11 (*p* < 0.01), 32 ± 11 (*p* < 0.05) and 46 ± 12 for miR-139-5p inhibition, miR-454-3p inhibition and negative controls, respectively (Figure 7E).

### 2.7. Overexpression of MiRs-139-5p and -454-3p Rescued ENG-knockdown-Induced HUVEC Dysfunction

**Figure 8:**
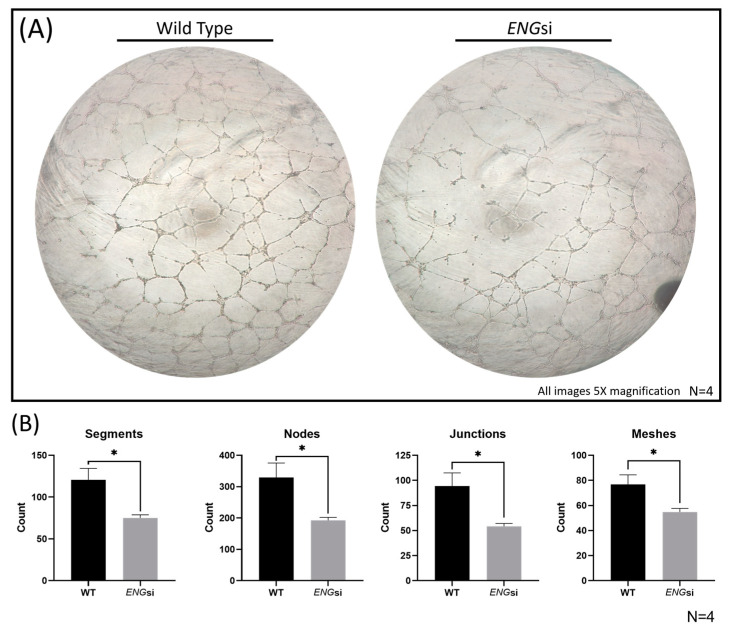
In vitro tube formation assay of wild-type (WT) HUVECs compared with that of *ENG*-knockdown (*ENG*si) HUVECs. (**A**) Images taken at 16 hours of WT and *ENG*si HUVEC tube formations (5× magnificaiton). (**B**) Significantly lower counts of segments, nodes, junctions and meshes were detected for HUVECs with *ENG*-knockdown (*ENG*si) compared with those in WT HUVECs (N = 4). * *p* < 0.05.

**Figure 9:**
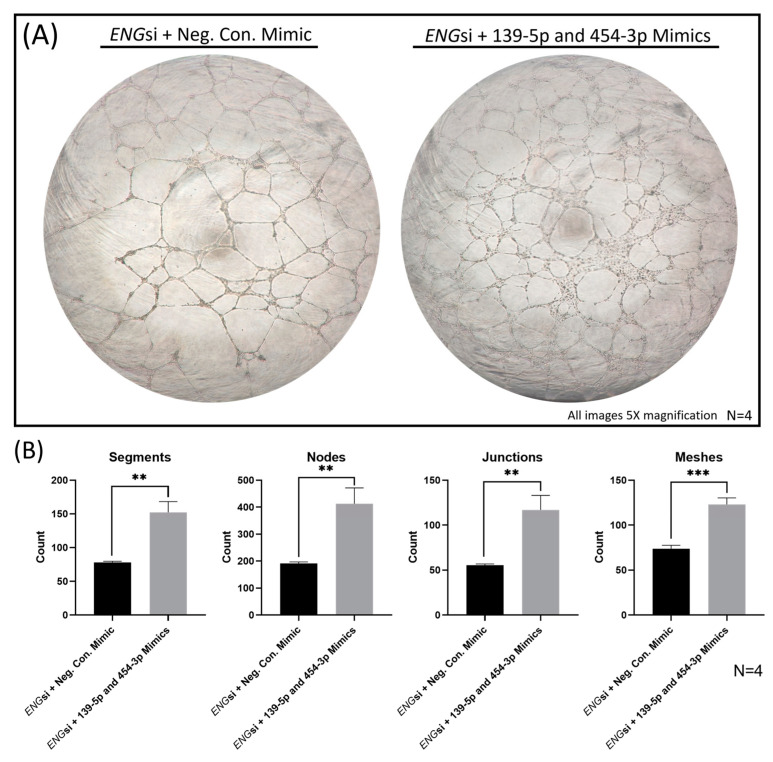
In vitro tube formation assay of *ENG*-knockdown (*ENG*si) HUVECs with either negative control mimics (Neg. Con. Mimic) or 139-5p/454-3p mimics. (**A**) Images of *ENG*si + Neg. Con. Mimics and *ENG*si + 139-5p and 454-3p mimics were taken at 16 hours (5× magnification). (**B**) Significantly increased counts of segments, nodes, junctions and meshes were detected in *ENG*-knockdown HUVECs with 139-5p/454-3p mimics compared with those in negative control mimics (N = 4). ** *p* < 0.01, *** *p* < 0.001.

Next, we sought to explore if the overexpression of miRs-139-5p and -454-3p could rescue *ENG*-knockdown-induced HUVEC dysfunction. Firstly, we confirmed that HUVECs with *ENG*-knockdown had a significant reduction in tube formation compared with that in HUVECs transfected with control siRNA (wild type, WT), shown in Figure 8. Segments, nodes, junctions and meshes were all significantly decreased (*p* < 0.05) in *ENG*si HUVECs (Figure 8B). For the functional rescue assay, miRs-139-5p and -454-3p were introduced into *ENG*si HUVECs by transfection with miR mimics (Supplementary Figure S4). Most notably, the simultaneous overexpression of miR-139-5p and miR-454-3p in *ENG*-knockdown HUVECs rescued HUVEC dysfunction, shown in Figure 9. Segments, nodes, junctions and meshes were all significantly increased in *ENG*-knockdown HUVECs with miR-139-5p/-454-3p mimics compared with those in negative control mimics (Figure 9B). 

## 3. Discussion

*ENG* mutations cause EC dysfunction, leading to HHT Type 1. Animal studies have identified ECs as the main pathological cell in HHT [36,40]. Inducible EC-specific *ENG* knockout in mice results in retinal AVMs and enlarged veins [36]. Garrido-Martin et al. demonstrated the formation of skin AVMs in EC-specific *ENG*-knockout mice following wounding [40]. The importance of *ENG* in EC function has also been highlighted in various EC models, including HUVECs and HHT-patient-derived BOECs, where loss of ENG resulted in enlarged or elongated morphology, dysregulated cellular proliferation, perturbed migration and polarity, and reduced tube formation [30,37,38,39,41,42]. Fernandez-L et al. showed that BOECs derived from HHT Type 1 patients demonstrate decreased tube formation and a disorganized actin cytoskeleton [39]. These researchers also demonstrated that the reduced ability of tube formation is correlated with reduced *ENG* expression in both HHT Type 1 and 2 BOECs [39]. The aim of this study was to explore the role of miRNAs in EC dysfunction caused by ENG depletion, which has rarely been documented. We performed a microarray assay to identify potentially dysregulated miRNAs for further analysis. Of the five miRNAs measured by RT-qPCR, miR-139-5p and miR-454-3p were found to be significantly downregulated in *ENG*-knockdown HUVECs, in line with the microarray data. The inhibition of miRs-139-5p or -454-3p reduced the angiogenic capacity of HUVECs as shown by a tube formation assay. Most notably, the overexpression of miRs-139-5p and -454-3p rescued *ENG*-knockdown-induced angiogenic dysfunction in HUVECs. These novel findings underscore the critical role miRs-139-5p and -454-3p may play in EC function and HHT pathogenesis.

A plethora of miRNAs have been identified as key regulators of EC function and ultimately, the angiogenic process. The EC-specific knockdown of Dicer, an endoribonuclease involved in miRNA biogenesis, resulted in the dysregulation of various EC-specific genes, including vascular endothelial growth factor (VEGF) receptor 2 and endothelial nitric oxide synthase [43]. The TGFβ/BMP signaling pathway has also been shown to both be regulated by and regulate various miRNAs in a multitude of cell types and disease states [44,45]. MiR-132 has been demonstrated to be upregulated during the inflammatory phase of wound healing in direct response to TGFβ1/2 and enhance the activation of TGFβ signaling by targeting Smad7 [46]. Previous work from our laboratory has demonstrated that circulating miR-210 may be a potential biomarker for the detection of untreated pulmonary AVMs [47]. We have also shown that miR-361-3p and -28-5p were significantly decreased in peripheral blood mononuclear cells (PBMCs), and miR-132-3p was downregulated in myeloid angiogenic cells from HHT patients [48,49]. Tabruyn et al. have shown that circulating miR-205 is significantly decreased and -27a significantly increased in HHT patients [50]. Recently, Ruiz-Llorente et al. identified circulating miR-370 and -10a as candidate biomarkers for the differentiation of HHT Type 1 and 2, respectively [51]. These data and the findings in the present study suggest that, apart from their widely studied role in cancer [52,53,54,55], miRNAs are involved in HHT pathogenesis and may also serve as biomarkers for HHT diagnosis.

MiR-139-5p (chromosomal locus 11q13.4) has been extensively studied in the diagnosis, prognosis and tumorigenesis of various cancers, including chronic myeloid leukemia, non-small cell lung carcinoma, prostate cancer, breast cancer and glioblastoma [53]. In tumorigenesis, miR-139-5p predominately acts as a tumor suppressor and is involved in PI3K/Akt, Wnt/β-catenin, RAS/MAPK and TGFβ/BMP signaling, to name a few [53]. The role of miR-139-5p in EC function has also been explored, albeit to a lesser extent. Similarly, miR-454-3p (chromosomal locus 17q22) has also been extensively studied in oncology [54,55,56] but to an even lesser extent in EC function. Previous studies have reported that these miRNAs are critical in EC function yet have demonstrated contradicting roles. Zhang et al. demonstrated that the inhibition of miR-139-5p suppresses VEGF-induced neovascularization of human microvascular endothelial cells by targeting phosphatase and tensin homolog (PTEN) [57]. Interestingly, they also found that miR-139-5p knockdown decreases cell viability and migration [57]. In contrast, Luo et al. reported that the inhibition of miR-139-5p in HUVECs and diabetic-derived BOECs results in increased tube formation by targeting c-Jun [58]. Similar to our findings, the authors also reported that the inhibition of miR-139-5p increases HUVEC migration [58]. Papangeli et al. showed increased HUVEC migration with miR-139-5p inhibition despite demonstrating that the intravenous administration of miR-139-5p inhibitors in the retina of a mouse model results in reduced vascularized area, radial expansion and branch points, corroborating our in vitro findings [59]. Li et al. found that miR-139-5p inhibition in primary endothelial cell cultures from pancreatic tumors results in reduced tube formation and migration [60].

There are few reports on the role of miR-454-3p in EC function. Xia et al. demonstrated that miR-454-3p inhibition results in increased HUVEC tube formation [55]. However, it is difficult to determine any specific effects of miR-454-3p inhibition since tube formation was conducted in the presence of a long non-coding RNA silencer and conditioned tumor medium. Liao et al. demonstrated that the inhibition of miR-454-3p reduces human aortic endothelial cell viability and increases apoptosis [61]. The variability seen in the literature regarding the role of the miRNAs implicated in EC dysfunction can be attributed to their diverse nature. MiRNAs are extremely context-specific and promiscuous biomolecules with tens to hundreds of targets. Their role in any context is dependent on their relative expression, the relative expression of their targets and the crosstalk of multiple regulatory pathways. Different culturing protocols, EC subtypes, experimental conditions and methodologies could all contribute to variable results. Despite the complexity of miRNA function, it is clear that miRs-139-5p and -454-3p play a critical role in EC function, where their differential expression leads to EC dysfunction. Limitations of this study include: (1) only one type of EC, i.e., HUVECs, was used, and (2) targets of miRs-139-5p and -454-3p were not explored.

To date, the role of any one miRNA in HHT pathogenesis has yet to be fully explored. The present study has demonstrated three novel findings: (1) *ENG*-knockdown HUVECs had a dysregulated miRNA profile, (2) miRs-139-5p and -454-3p were found to be significantly decreased in *ENG*-knockdown HUVECs, and (3) miRs-139-5p and -454-3p were critical for normal HUVEC function. Most importantly, we have shown that the inhibition of miR-139-5p or miR-454-3p resulted in angiogenic dysfunction similar to that shown in HHT Type 1 BOECs and that the overexpression of these miRNAs rescued *ENG*-knockdown-induced HUVEC dysfunction. The downregulation of miRs-139-5p and -454-3p in *ENG*-knockdown endothelial cells potentially serves as a novel mechanism in HHT pathogenesis and may be crucial in the identification of novel therapeutic targets. Further research is necessary to understand the role of miRNAs in HHT pathogenesis.

## 4. Materials and Methods

### 4.1. Culture of HUVECs

HUVECs purchased from Lonza (Walkersville, MD, USA) were cultured in fibronectin-coated (10 μg/mL) T25 or T75 flasks at 37 °C and 5% CO_2_. HUVECs were maintained in complete Endothelial Cell Growth Medium-2 (EGM-2) (Lonza, EGM-2 BulletKit, cat# CC-3162) supplemented with 5% fetal bovine serum (FBS). Cells at 80–90% confluence and ≤ to the fifth passage were used for all experiments. HUVECs were detached with trypsin-EDTA (Multicell Trypsin/EDTA 0.05% trypsin, 0.53 mM EDTA with sodium bicarbonate). HUVECs obtained for this study were tested negative for mycoplasma, bacteria, viruses and fungi by the supplier, and used in low passage.

### 4.2. ENG Short Interfering RNA (siRNA) Transfection

This protocol was adapted from our previous work [35]. Six-well plates were seeded with HUVECs (1.5 × 10^5^ cells/well) and cultured until 60–80% confluence (approx. 24 h). At this point, negative control siRNA- or *ENG* siRNA-Lipofectamine RNAiMAX complexes were added to each well of cells. The complexes were prepared as follows: 3 μL of 10 μM siRNA (*ENG* or negative control) and 5 μL of Lipofectamine RNAiMAX were diluted in 250 μL of Opti-MEM reduced serum medium, respectively. These diluted mixes were then combined and incubated for 15 min to allow for complex formation. The complexes were added to cells with 2.5 mL of fresh complete EGM-2 medium (final siRNA concentration: 10 nM). The cells were cultured in the media–complex mixture for 48 h and then used for experimentation. Lipofectamine RNAiMAX (cat# 13778075), Opti-MEM (cat# 31985062), Silencer Select Negative Control No. 2 siRNA (cat# 4390846) and *ENG* siRNA (cat# 4392420, siRNA ID s4679) were purchased from Thermo Fisher Scientific (Burlington, ON, Canada).

### 4.3. Western Blotting

Cellular protein was isolated from HUVECs in RIPA Lysis Buffer (25 mM TrisHCl pH 7.6, 150 mM NaCl, 1% NP-40, 1% sodium deoxycholate, 0.1% SDS) supplemented with a 100× Halt Protease Phosphatase Inhibitor Cocktail (1:100 dilution, Sigma, Oakville, ON, Canada). Protein concentration was measured via a Bradford assay. Protein separation (50 μg/lane) was conducted by SDS-PAGE (4 to 12% Tris-Glycine gel) and electrically transferred onto a 0.2 μm nitrocellulose membrane. The membrane was blocked in 1× TBST (50 mM TrisHCl, 150 mM NaCl, pH 7.5, 0.1% Tween-20) containing 5% skim milk for 1 h at room temperature followed by primary antibody incubation overnight at 4 °C (ENG, 1:1000 dilution; β-actin, 1:10,000 dilution). After incubation, the membrane was washed twice with 1× TBST for 5 min. The membrane was incubated with secondary antibodies (1:5000 dilution) in a blocking buffer (1× TBST containing 5% skim milk) for 1 h in the dark at room temperature. After incubation, the membranes were washed 3 times with 1× TBST for 15 min. Protein bands were visualized with the Odyssey fluorescence imaging system (LI-COR Biosciences, Lincoln, NE, USA). Densitometry analysis was performed with Image Studio Lite Quantification Software 5.0 (LI-COR Biosciences). β-actin was used as an internal loading control. Primary antibodies used: ENG, Cell Signalling Technologies (Oakville, ON, Canada), mouse, cat# 14606S, clone 3A9 and β-actin, ABclonal Technology (Woburn, MA, USA), rabbit, cat# AC026. Secondary antibodies used: Goat anti-mouse, Thermo Fisher Scientific, cat# A32742 and goat anti-rabbit, LI-COR Biosciences, cat# 926-32211.

### 4.4. Total RNA Isolation from HUVECs

Total RNA was isolated from HUVECs with a Qiagen RNeasy Mini Kit (cat# 217004, Qiagen, Toronto, ON, Canada). Cells in a well of a 6-well plate were scraped in 700 μL of Qiazol lysis reagent and transferred to a 1.5 mL Eppendorf tube. The mixture was incubated for 5 min at room temperature. After incubation, 140 μL of chloroform was added and shaken vigorously for 15 s. The mixture was incubated at room temperature for 3 min and centrifuged at 12,000× *g* for 15 min at 4 °C. After centrifugation, 300 μL of the supernatant was carefully extracted without disruption of the interphase. Subsequently, 1.5 volumes or 450 μL of 100% ethanol was added and mixed thoroughly by pipetting. To capture the RNA, 700 μL of this mixture was added to an RNeasy Mini column and centrifuged at 10,000× *g* for 15 s at 4 °C. The column was then washed with 500 μL of Buffer RPE at 10,000× *g* for 15 s at 4 °C. This was repeated with a 2 min centrifugation. Then, the column was washed with 100% ethanol and centrifuged for 1 min at 10,000× *g* at 4 °C. Finally, RNA was eluted with 30 μL of RNase-free water at 10,000× *g* for 1 min at 4 °C. A NanoDrop 2000 spectrophotometer was used to assess the concentration and quality of RNA before storage at −80 °C. All components used were DNase, RNase and pyrogen-free.

### 4.5. MiRNA Microarray of Total RNA from HUVECs

To profile miRNAs in HUVECs, a TaqMan Low-Density MicroRNA Microarray (Thermo Fisher Scientific, Card A v2.0, cat# 4398965) covering 377 human miRNAs was used. Total RNA samples were reverse-transcribed into cDNA with a TaqMan MicroRNA Reverse Transcription (RT) Kit (cat# 4366596) and Megaplex RT Primers. The total volume of the RT reaction was 7.5 μL and comprised 3 μL of total RNA (600 ng), 0.8 μL of Megaplex RT primers (10×), 0.2 μL of dNTPs (100 mmol/L), 1.5 μL of MultiScribe Reverse Transcriptase (50 U/μL), 0.8 μL of 10× RT buffer, 0.9 μL of MgCl_2_ (25 mmol/L), 0.1 μL of RNase inhibitor (20 U/μL) and 0.2 μL of nuclease-free water. The thermocycling protocol for RT was performed as follows: 40 cycles of 16 °C for 2 min, 42 °C for 1 min, and 50 °C for 1 s followed by 85 °C for 5 min and 4 °C hold. The total reaction volume for PCR was 900 μL and comprised 450 μL of 2× TaqMan Universal PCR Master Mix (no AmpErase UNG), 444 μL of nuclease-free water and 6 μL of Megaplex RT product. Then, 100 μL of diluted RT product was dispensed in each of the 8 ports of the Array Card A v2.0. The card was sealed and centrifuged twice at 1200 rpm for 1 min. PCR was performed on a ViiA 7 Real-Time PCR System (Applied Biosystems, Waltham, MA, USA) with a 384-well TaqMan Low-Density Array block. The results were analyzed using RQ Study software 1.4 (Thermo Fisher Scientific) and normalized to U6 snRNA as determined by the NormFinder Excel plugin (https://moma.dk/normfinder-software, access date: 23 February 2021). Select miRNAs of interest were determined based on a fold change of 1.5 or greater and <30 Ct value [62,63].

### 4.6. Reverse Transcription Quantitative Polymerase Chain Reaction (RT-qPCR) for MiRNA Validation

As RT for microarray analysis was completed in a single reaction tube using pooled primers (https://assets.fishersci.com/TFS-Assets/LSG/manuals/cms_054742.pdf, access date: 9 December 2022) that may result in mis-priming between two different primers or between a primer and an RNA template, reducing the accuracy of array results, RT-qPCR was performed to measure each select miRNA to validate the array data. The RT reaction was carried out with a total volume of 15 μL consisting of 7 μL of RT master mix, 3 μL of 5× RT primer and 5 μL of RNA sample (total RNA 20 ng). For each reaction, the RT master mix was prepared as follows: 0.15 μL of 100 mM dNTPs, 1 μL of (50 U/μL) MultiScribe Reverse Transcriptase, 1.5 μL of 10× reverse transcription buffer, 0.19 μL of (20 U/μL) RNase inhibitor and 4.16 μL of nuclease-free water. RT was performed on a Veriti 96-well thermal cycler according to the following protocol: 16 °C for 30 min, 42 °C for 30 min, 85 °C for 5 min and 4 °C hold. PCR was performed in a total volume of 10 μL consisting of 1 μL of RT product, 0.5 ul of 20× miRNA PCR primer, 5 μL of 2×TaqMan Fast Advanced Master Mix and 3.5 μL of nuclease-free water. PCR was performed on a QuantStudio 7 Flex Real-Time PCR System (Thermo Fisher Scientific) according to the following thermocycling protocol: 95 °C for 20 s and 40 cycles of 95 °C for 1 s and 60 °C for 20 s. Relative quantification of the expression of individual miRNAs against endogenous U6 snRNA was carried out.

### 4.7. MirVana miRNA Inhibitor or Mimic Transfection

For miRNA inhibitor transfection, six-well plates were seeded with HUVECs (1.5 × 105 cells/well) and cultured until 60–80% confluence (approx. 24 h). MirVana miRNA inhibitors (Thermo Fisher Scientific, cat# 4464084; hsa-miR-139-5p, Assay ID MH11749; hsa-miR-454-3p, Assay ID MH12343) and MiRNA Negative Control #1 (Thermo Fisher Scientific, cat# 4464076) were thawed on ice and spun down before use. MiRNA inhibitor/transfection reagent complexes were generated as follows: 5 μL of Lipofectamine RNAiMAX and 5 μL of 10 μM miRNA inhibitor or negative control were diluted in 125 μL of Opti-MEM reduced serum medium, separately. The diluted reagent and inhibitor were then mixed, incubated for 5 min at room temperature and added to cells replenished with 1.75 mL of fresh complete EGM-2 medium (final miRNA inhibitor concentration: 25 nM). The cells were cultured for 24 h and then used for experimentation. For miRNA mimic transfection, HUVECs were transfected with *ENG* siRNA and cultured for 24 h as described above. Subsequently, 5 μL of mimics (2.5 μL of 10 μM miR-139-5p plus 2.5 μL of 10 μM miR-454-3p) or 5 μL of miRNA-negative control was used for transfection of *ENG*-knockdown HUVECs, which was done in the same manner as with miRNA inhibitors. The cells were cultured for another 24 h after mimic transfection for the tube formation assay.

### 4.8. Cell Viability and Proliferation Assay

A cell-counting kit-8 (CCK8/WST-8) assay was used for the measurement of cellular viability and proliferation (Sigma, cat# 96992). Ninety-six-well plates were seeded with HUVECs (5 × 10^3^ cells/well for viability, and 2.5 × 10^3^ cells/well for proliferation). The cells were cultured for 24 h in complete EGM-2 medium and then transfected as described above. After transfection, the cells were either supplemented with fresh, complete EGM-2 medium (proliferation) or endothelial basal medium-2 (EBM-2) without serum (viability) and cultured for 24 h. WST-8 was then added, and the cells were incubated for 4 h at 37 °C and 5% CO_2_. Subsequently, the absorbance (450 nm) was measured with a microplate reader (endpoint analysis) and compared between different groups of cells. All conditions were carried out in triplicate.

### 4.9. Apoptosis Assay

Apoptosis was measured via flow cytometric detection of Annexin V (AV). HUVECs were transfected with MirVana miRNA Inhibitors and Negative Control #1 as described above. Appropriate fluorescence controls were used, including unstained and double-stained (AV and propidium iodide (PI)) negative controls (healthy cells) and unstained, single-stained (AV or PI) and double-stained positive controls (Supplementary Figure S1). Positive controls were generated with hydrogen peroxide (10 mM H_2_O_2_) treatment for 1–2 h at 37 °C and 5% CO_2_. The cells were detached with PBS containing 1 mM EDTA for analysis. Positive controls were spiked with negative controls (approx. 50% cell viability) to ensure a background level of healthy cells for appropriate gating. The cells were resuspended at a concentration of 1 × 10^3^ cells/μL in cold 1× Annexin V Binding Buffer (BD Pharmingen, Mississauga, ON, Canada, cat# 51-66121E). Then, 100 μL of each cell suspension was incubated with either 0.5 μL of Alexa Fluor (APC channel) AV (BioLegend, San Diego, CA, USA, cat# 640911), 1.5 μL of PI (PE-CF594-A channel) (BD Pharmingen, cat# 51-66211E) or both at room temperature for 15 min in the dark. After incubation, the cells were put on ice and supplemented with 200 μL of cold 1× Annexin V Binding Buffer (final volume of 300 μL). The cells were analyzed via flow cytometry immediately after staining on a BD LSRFortessa X-20 Cell Analyzer with BD FACSDiva Software 6.1.2. Analysis of flow cytometric data was conducted with FlowJo 10.8.1 software. Forward/side scatter scatterplots were used to exclude cellular debris and doublets of 1 × 10^4^ recorded events.

### 4.10. Cellular Migration Assay

The cellular migration assay was performed with an Ibidi wound healing assay (culture-insert 2 well, Ibidi, Martinsried, Germany, cat# 81176) in a 12-well plate. HUVECs were cultured, transfected and detached as described above. Ibidi 2-well culture inserts were placed in the center of the well with slight pressure to ensure adhesion. In each well of the Ibidi 2-well culture inserts, 70 μL (28,000 cells) of cells was seeded. The cells were incubated at 37 °C and 5% CO_2_ for 24 h with fresh, complete EGM-2 medium until confluent. The plate was handled carefully to prevent shaking. The cells were then serum-starved (EBM-2 medium, 0.5% FBS) for 4 h, and the insert was gently removed with sterile tweezers to create an open wound area. The cells were washed with warm phosphate-buffered saline (PBS) and replenished with serum-reduced EBM-2 medium (0.5% FBS). Cell migration was carried out at 37 °C under an atmosphere of 5% CO_2_ and live-imaged for 12 h with a Hamamatsu Camera and a Zeiss Axio Observer microscope (10× magnification) with Zen Pro 3.6 software (Zeiss Canada Ltd., Toronto, Canada). The images were processed with Zen 3.3 Lite (Blue Edition) and analyzed with TScratch 1.0. The percent of the open wound area of 5 fields was calculated and averaged. All conditions were done in duplicate.

### 4.11. Tube Formation Assay

Geltrex LDEV-free reduced growth factor basement membrane matrix (Gibco, Burlington, ON, Canada, cat# A1413201) was used. The Geltrex basement membrane matrix was thawed overnight in a 4 °C fridge. Once thawed, it was mixed well and aliquoted (80 μL/well) into a 96-well plate (on ice) with chilled pipette tips. To prevent air bubble formation, the basement membrane was dispensed without a full stop, and the plate was centrifuged at 300× *g* for 10 min at 4 °C. Then, the coated plate was incubated at 37 °C and 5% CO_2_ for 30 min. HUVECs were cultured and transfected as described above. The cells were serum-starved for 4 h and detached with trypsin-EDTA, then resuspended at 2 × 10^5^ cells/mL in complete EGM-2 medium. Each coated well received 100 μL of cell suspension (2 × 10^4^ cells/well). Tube formation was carried out at 37 °C under an atmosphere of 5% CO_2_ and live-imaged with a Hamamatsu Camera and a Zeiss Axio Observer microscope (5× magnification) with Zen Pro 3.6 software. The images were processed at the 16 h time point with Zen 3.3 Lite (Blue Edition) and analyzed with Angiogenesis Analyzer 1.0 software for ImageJ2 or Fiji. All conditions were done in duplicate.

### 4.12. Statistical Analysis

All data were normally distributed as determined by the Shapiro–Wilk test and expressed as the mean ± standard deviation (SD). Statistical analyses were performed with an unpaired two-tailed Student’s *t*-test with Welch’s correction using GraphPad Prism 9. For comparison between three groups, a one-way ANOVA with Tukey’s multiple comparison test was conducted. *p* < 0.05 was considered statistically significant.

## Figures and Tables

**Table 1:** Dysregulated miRNAs identified by the microarray analysis after systematic exclusion.

MicroRNAs	Accession	Assay Target Sequence	Fold Decrease
hsa-miR-16-5p	MIMAT0000069	UAGCAGCACGUAAAUAUUGGCG	1.8 ± 0.43
hsa-miR-17-5p	MIMAT0000070	CAAAGUGCUUACAGUGCAGGUAG	1.6 ± 0.11
hsa-miR-19b-3p	MIMAT0000074	UGUGCAAAUCCAUGCAAAACUGA	1.7 ± 0.55
hsa-miR-20a-5p	MIMAT0000075	UAAAGUGCUUAUAGUGCAGGUAG	1.6 ± 0.57
hsa-miR-21-5p	MIMAT0000076	UAGCUUAUCAGACUGAUGUUGA	2.4 ± 0.88
hsa-miR-24-3p	MIMAT0000080	UGGCUCAGUUCAGCAGGAACAG	2.0 ± 1.16
hsa-miR-26a-5p	MIMAT0000082	UUCAAGUAAUCCAGGAUAGGCU	1.7 ± 0.45
hsa-miR-28-3p	MIMAT0004502	CACUAGAUUGUGAGCUCCUGGA	1.7 ± 0.45
hsa-miR-29a-3p	MIMAT0000086	UAGCACCAUCUGAAAUCGGUUA	2.0 ± 0.51
hsa-miR-30b-5p	MIMAT0000420	UGUAAACAUCCUACACUCAGCU	1.7 ± 0.46
hsa-miR-30c-5p	MIMAT0000244	UGUAAACAUCCUACACUCUCAGC	1.9 ± 0.68
hsa-miR-31-5p	MIMAT0000089	AGGCAAGAUGCUGGCAUAGCU	1.9 ± 0.90
hsa-miR-99a-5p	MIMAT0000097	AACCCGUAGAUCCGAUCUUGUG	2.0 ± 0.68
hsa-miR-99b-5p	MIMAT0000689	CACCCGUAGAACCGACCUUGCG	4.6 ± 1.64
hsa-miR-125b-5p	MIMAT0000423	UCCCUGAGACCCUAACUUGUGA	1.8 ± 0.64
hsa-miR-126-3p	MIMAT0000445	UCGUACCGUGAGUAAUAAUGCG	2.0 ± 0.37
hsa-miR-139-5p	MIMAT0000250	UCUACAGUGCACGUGUCUCCAG	2.1 ± 0.40
hsa-miR-146a-5p	MIMAT0000449	UGAGAACUGAAUUCCAUGGGUU	1.9 ± 0.49
hsa-miR-186-5p	MIMAT0000456	CAAAGAAUUCUCCUUUUGGGCU	2.5 ± 0.70
hsa-miR-191-5p	MIMAT0000440	CAACGGAAUCCCAAAAGCAGCUG	1.7 ± 0.33
hsa-miR-193b-3p	MIMAT0002819	AACUGGCCCUCAAAGUCCCGCU	2.0 ± 0.56
hsa-miR-218-5p	MIMAT0000275	UUGUGCUUGAUCUAACCAUGU	1.7 ± 0.46
hsa-miR-222-3p	MIMAT0000279	AGCUACAUCUGGCUACUGGGU	1.7 ± 0.39
hsa-miR-320a	MI0000542	AAAAGCUGGGUUGAGAGGGCGA	1.6 ± 0.26
hsa-let-7b-5p	MIMAT0000063	UGAGGUAGUAGGUUGUGUGGUU	3.3 ± 0.60
hsa-miR-324-3p	MIMAT0000762	ACUGCCCCAGGUGCUGCUGG	2.1 ± 0.41
hsa-miR-345-5p	MIMAT0000772	GCUGACUCCUAGUCCAGGGCUC	2.0 ± 0.73
hsa-miR-454-3p	MIMAT0003885	UAGUGCAAUAUUGCUUAUAGGGU	1.5 ± 0.09
hsa-miR-484	MIMAT0002174	UCAGGCUCAGUCCCCUCCCGAU	1.5 ± 0.16
hsa-miR-574-3p	MIMAT0003239	CACGCUCAUGCACACACCCACA	1.7 ± 0.22
hsa-miR-590-5p	MIMAT0003258	GAGCUUAUUCAUAAAAGUGCAG	1.6 ± 0.12
hsa-miR-376c-3p	MIMAT0000720	AACAUAGAGGAAAUUCCACGU	2.0 ± 0.29

The fold decrease is from three independent microarray analyses.

## Data Availability

Data is contained within this article or Supplementary Material.

## Supplementary Materials

The following supporting information can be downloaded at: https://www.mdpi.com/article/10.3390/ijms24054916/s1.
