# Supplementary material for: The Potential Role of MiRs-139-5p and -454-3p in Endoglin-Knockdown-Induced Angiogenic Dysfunction in HUVECs
Source: Int J Mol Sci. 2023 Mar 3;24(5):4916. doi: 10.3390/ijms24054916 (PMC10003543; doi:10.3390/ijms24054916)
Supplement: Supplementary file 1 [file ijms-24-04916-s001.zip › ijms-2128440-supplementary.pdf]

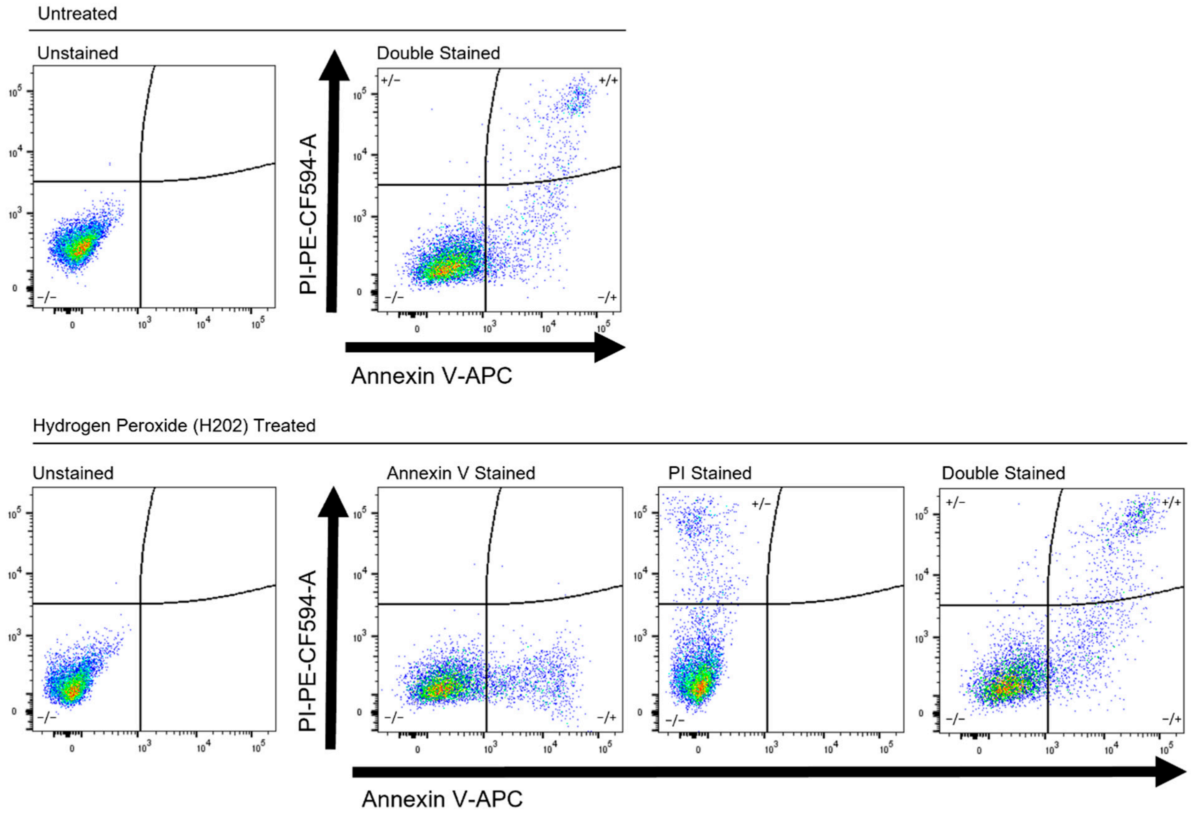

Figure S1. Fluorescence controls used for the flow cytometric detection of Annexin V and PI in HUVECs.

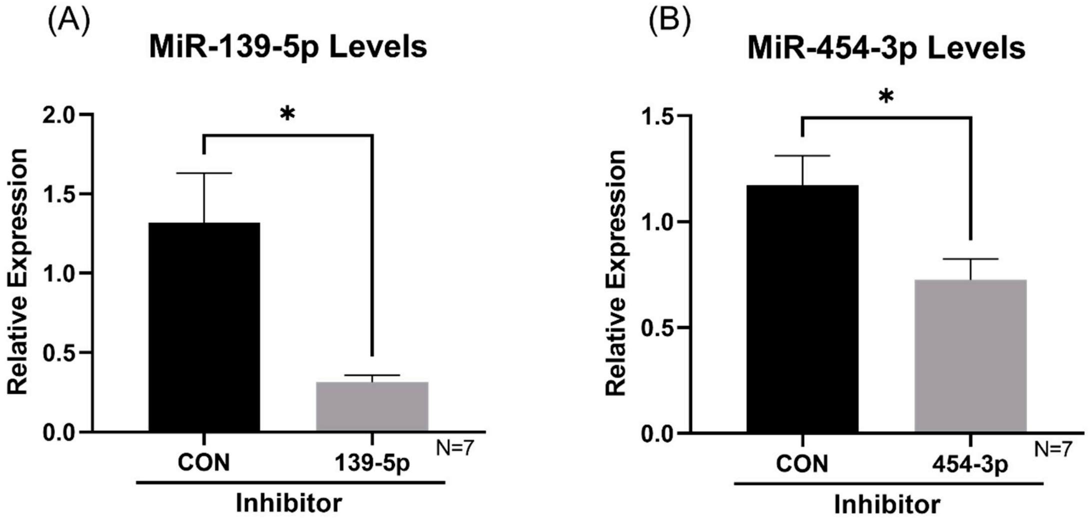

Figure S2. RT-qPCR measurement of miRs-139-5p and -454-3p after transfection with miRNA inhibitors. (**A**) Transfection of HUVECs with a miR-139-5p inhibitor resulted in significantly lower expression of miR-139-5p compared with negative controls (CON). (**B**) Transfection of HUVECs with a miR-454-3p inhibitor resulted in significantly lower levels of miR-454-3p compared with negative controls. *$p < 0.05$.

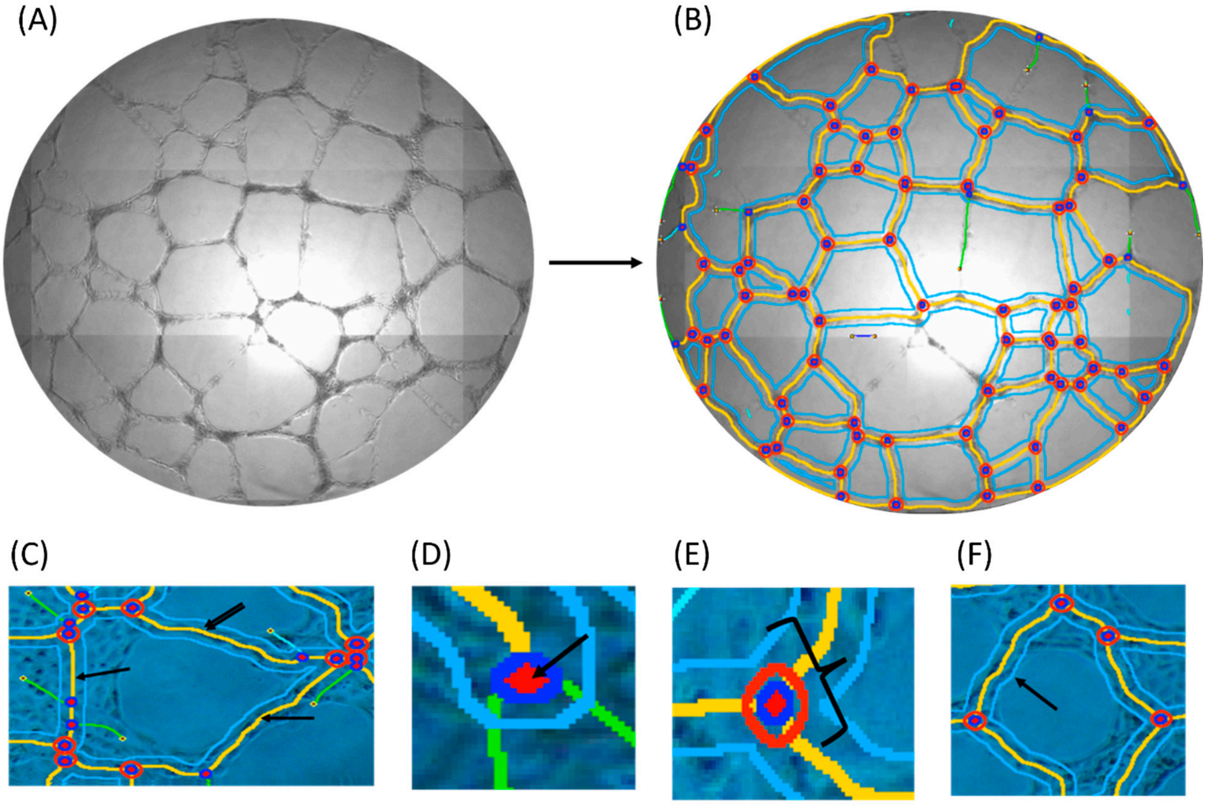

Figure S3. Tube formation analysis using the Angiogenesis Analyzer Software for ImageJ. (**A**) A standard tube formation image used for analysis. Images were cropped to fit a circle to remove the plate well periphery that could be misinterpreted by Angiogenesis Analyzer software. (**B**) An image after Angiogenesis Analyzer software labels tube-like structures. (**C**) Segments are denoted as yellow lines (black arrows) and are defined as a tube network connected by two junctions. (**D**) Nodes are shown as red pixels (black arrow) and are defined as a central connecting point. (**E**) Junctions are depicted as dark blue around red pixels (black right brace) and are defined as a central connecting point (node) with 3 connections (segments). (**F**) Meshes or rings are denoted as sky blue (black arrow) and are defined as areas enclosed by segments.

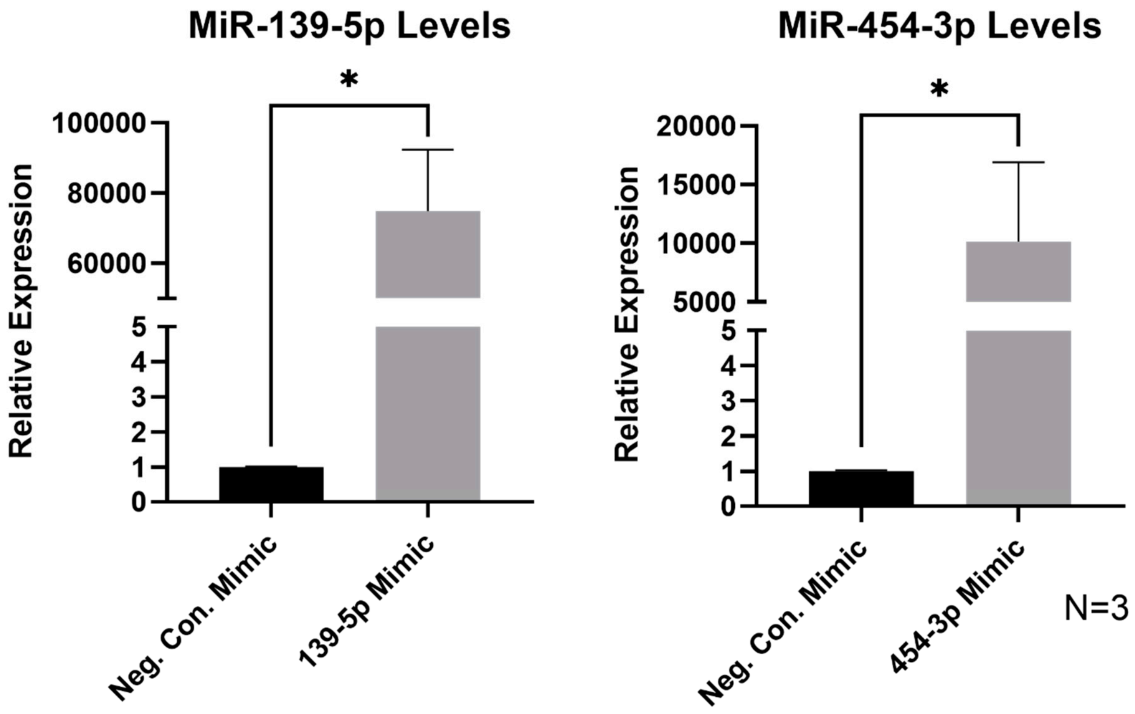

Figure S4: Measurement of miRs-139-5p and -454-3p by RT-qPCR after transfection of *ENG*-knockdown HUVECs with miRNA mimics. As shown in this figure, simultaneous transfection of *ENG*-knockdown HUVECs with miR-139-5p and miR-454-3p mimics resulted in significantly higher expression of both miRNAs compared with negative control mimics (Neg. Con. Mimic). $*p < 0.05$.
